# Practical Departments

**Published:** 1905-10-28

**Authors:** 


### PRACTICAL DEPARTMENTS.

### MESSRS. ROYLES' HEATING APPARATUS.

Messrs. Royles, of Irlam o' th' Heights, near Manchester, are another firm who have been engaged in the manufacture and fixing of heating apparatus for a great many years. They are known principally for their "Calorifiers," the barbarous term that heating and ventilating engineers have applied to an apparatus which converts the latent heat of steam into the visible heat of hot water, in a cylinder in which there is a nest of tubes, through which the water is be heated passes, while the steam passes in the space surrounding it. Messrs. Royle have a special form of tube for the water, which may best be described as an ordinary tube flattened or indented, at regular intervals, on two opposite faces, and again flattened on two opposite faces at right angles to the first two, in the intervals between the first indentations. It is claimed that the arrangement gives a larger surface across which the heat from the steam can pass to the water, that the water is thoroughly broken up by the irregularities of the tubes, and in addition the tubes are flexible, and allow for the expansions and contractions that take place when the apparatus is at different temperatures. It is made up in different forms, called Feed Water Heaters, where it is intended for heating the water for a steam boiler, and Calorifiers where it is intended for heating-water for radiators, hot-water supply, or similar work. In connection with the latter, an automatic governor is supplied, consisting of a bar of metal stretched between the top and bottom of the apparatus, and against which the steam entry valve impinges. As the temperature of the whole apparatus rises, it expands, straightening the metal bar with it, and bringing it closer to the steam valve, which is gradually forced inwards, and is as gradually released when the temperature falls, so that more steam may be admitted, the admission of steam to the cylinder being controlled in this way. Storage Calorifiers are also made by the firm on the same lines, designed to provide a store of hot water, for contingencies, and also for economy, so that the steam that is being made by the boiler during those hours when there is not much call for hot water shall not be wasted. Amongst other apparatus of interest made by the firm is an automatic Water Blender (Buley's Patent), for warm baths, or any service where it is required to keep the inflow of water at an even temperature. It consists of a cylinder in which the hot and cold water mix, having an inner cylinder of thin copper, one end of which is mechanically in connection with the valve controlling the hot-water supply. The mechanical connection can be adjusted so that any desired temperature may be obtained. The copper cylinder contains air, which expanding, or contracting, as the temperature of the mixture surrounding it rises or falls, acts on the valve mechanism, lessening or increasing the supply of hot water as required. Messrs. Royle make other apparatus connected with heating, such as evaporators and condensers for producing pure water, steam kettles for quickly boiling water by steam, radiators with the indented tubes described above, gas kettles for heating water by gas, steam ovens, steam traps and separators, and other important accessories where economy is desirable in steam plant.

